# P2X7 Receptor in Bone Marrow-Derived Cells Aggravates Tuberculosis Caused by Hypervirulent *Mycobacterium bovis*

**DOI:** 10.3389/fimmu.2017.00435

**Published:** 2017-04-13

**Authors:** Caio César Barbosa Bomfim, Eduardo Pinheiro Amaral, Alexandra dos Anjos Cassado, Érika Machado Salles, Rogério Silva do Nascimento, Elena Lasunskaia, Mario Hiroyuki Hirata, José Maria Álvarez, Maria Regina D’Império-Lima

**Affiliations:** ^1^Department of Immunology, Biomedical Science Institute, University of São Paulo (USP), São Paulo, Brazil; ^2^Laboratory of Biology of Recognition, State University of North Fluminense, Campos dos Goytacazes, Brazil; ^3^Faculty of Pharmaceutical Sciences, Department of Clinical Chemistry and Toxicology, University of São Paulo (USP), São Paulo, Brazil

**Keywords:** tuberculosis, hypervirulent mycobacteria, P2X7 receptor, bone marrow-derived cells, mouse models

## Abstract

Tuberculosis (TB) remains a serious public health problem despite the great scientific advances in the recent decades. We have previously shown that aggressive forms of TB caused by hypervirulent strains of *Mycobacterium tuberculosis* and *Mycobacterium bovis* are attenuated in mice lacking the P2X7 receptor, an ion channel activated by extracellular ATP. Therefore, P2X7 receptor is a potential target for therapeutic intervention. *In vitro*, hypervirulent mycobacteria cause macrophage death by a P2X7-dependent mechanism that facilitates bacillus dissemination. However, as P2X7 receptor is expressed in both bone marrow (BM)-derived cells and lung structural cells, several cellular mechanisms can operate *in vivo*. To investigate whether the presence of P2X7 receptor in BM-derived cells contributes to TB severity, we generated chimeric mice by adoptive transfer of hematopoietic cells from C57BL/6 or P2X7^−/−^ mice into CD45.1 irradiated mice. After infection with hypervirulent mycobacteria (MP287/03 strain of *M. bovis*), P2X7^−/−^>CD45.1 mice recapitulated the TB resistance observed in P2X7^−/−^ mice. These chimeric mice showed lower lung bacterial load and attenuated pneumonia compared to C57BL/6>CD45.1 mice. Lung necrosis and bacterial dissemination to the spleen and liver were also reduced in P2X7^−/−^>CD45.1 mice compared to C57BL/6>CD45.1 mice. Furthermore, an immature-like myeloid cell population showing a Ly6G^int^ phenotype was observed in the lungs of infected C57BL/6 and C57BL/6>CD45.1 mice, whereas P2X7^−/−^ and P2X7^−/−^>CD45.1 mice showed a typical neutrophil (Ly6G^hi^) population. This study clearly demonstrates that P2X7 receptor in BM-derived cells plays a critical role in the progression of severe TB.

## Introduction

Nearly a quarter of the global population harbors bacteria of the *Mycobacterium tuberculosis* complex, resulting in an estimated 10.4 million new cases of active tuberculosis (TB) in 2015 (1, 2). Infection typically occurs when an individual inhales aerosolized droplets containing the mycobacteria (3). In the pulmonary alveoli, the mycobacteria may be ingested by alveolar macrophages that recruit inflammatory cells (4). Surviving bacilli multiply within the macrophage and, in most cases, are trapped inside primary granulomas. The equilibrium between host defense and the mycobacteria leads to latent infection. Active TB can develop through progression of recently acquired infection (primary disease) or reactivation of latent infection. Around 10% of active TB cases are due to progressive primary TB, which is an aggressive form of the illness that affect mostly immunodeficient patients and children under 5 years (5). The rates of latent TB reactivation range from 3 to 10% per lifespan in immunocompetent patients and increase markedly in immunodeficient patients (6–8). By promoting a progressive decline in cell-mediated immunity, co-infection with human immunodeficiency virus (HIV) greatly enhances TB incidence and severity. HIV co-infection was reported in 1.2 million (11%) of the people who developed TB in 2014 (1). Therefore, TB is the leading cause of death among individuals with acquired immunodeficiency syndrome (9, 10).

Severe TB cases are distinguished by the fast increase of granulomatous infiltrates that result in tuberculous pneumonia and, eventually, in hematogenous bacillus dissemination, such as in the miliary form of the disease. A hallmark of the serious illness is the existence of pulmonary caseous granulomas in which a central necrotic lesion contains many extracellular mycobacteria (11). Intense necrotic death of macrophages seems to result from the failure of host immune response to control bacillus growth. Consequently, the respiratory function is affected by the extensive tissue injury and causes the patient death. Therefore, many efforts have been made to elucidate how macrophages die following mycobacterial infection (12). One of the main difficulties to understand the pathogenesis of severe TB was the lack of animal models that develop pulmonary necrotic granulomas, as these lesions are unusual in murine models of TB, such as infection with mycobacteria of the virulent H37Rv strain. Therefore, our research group has established murine models in which C57BL/6 mice are infected with a low dose of hypervirulent mycobacteria (13, 14). Hypervirulent Beijing 1471 *M. tuberculosis* strain and MP287/03 *Mycobacterium bovis* strain induce extensive pulmonary inflammation, necrosis, high bacillus dissemination, and mouse death (13). These experimental models were used to determine whether the recognition of damage signals modulates the disease.

During necrotic cell death, ATP is released in the extracellular environment (15–17). Extracellular ATP (eATP) is a damage signal that is recognized by many cell types through different P2 purinergic receptors. Among them, the P2X7 receptor leads to release of proinflammatory cytokines and induces cell death (18). This molecule is a ligand-gated ion channel that is activated by high eATP concentrations, a characteristic of extensive tissue injury (18, 19). P2X7 engagement causes changes in intracellular ion balance that promotes the NLRP3 inflammasome activation and secretion of active IL-1β and IL-18, as well as cell death by pyroptosis (20). Furthermore, the stimulation of P2X7 receptor induces the opening of large pores in the plasma membrane, which allows the free flow of macromolecules. The duration and intensity of the stimulus establish whether P2X7 receptor activation promotes cell necrosis or apoptosis (21). By examining TB progression in mice deficient in P2X7 receptor that were infected with H37Rv, Beijing 1471, and MP287/03 bacilli, we demonstrated that the crucial role of P2X7 receptor in the aggressive forms of the disease (13). These mice showed increased resistance to infection evidenced by diminished bacterial load in the lungs, liver, and spleen. The lack of P2X7 receptor also caused reductions of inflammatory cellular infiltrate and tissue necrosis in the lung, which corroborated our hypothesis of the involvement of damage signals in the pathogenesis of severe TB.

To determine the mechanism involved in the deleterious role of P2X7 receptor in severe TB, we performed *in vitro* experiments using bone marrow (BM)-derived macrophages. We observed that eATP induces the P2X7-mediated killing of intracellular H37Rv bacilli and the P2X7-mediated release of viable hypervirulent Beijing 1471 and MP287/03 bacilli (13). Although this finding suggests that P2X7 signaling in infected macrophages facilitates the dissemination of hypervirulent mycobacteria, several other mechanisms might also operate *in vivo* because this receptor is expressed in many BM-derived cells and structural cells of the lungs, such as vascular endothelial cells, alveolar epithelial type I cells, and fibroblasts (22–25). Therefore, in the present study, we sought to investigate *in vivo* whether P2X7 receptor in BM-derived cells contributes to TB severity. Clarifying this issue may help understand the pathophysiology of aggressive forms of TB and give the theoretical background to develop new therapeutic approaches to ameliorate the outcome of the disease.

## Results

### P2X7 Receptor in BM-Derived Cells Increases Lung Weight, Lung Relative Mass, and Cellularity in Severe TB

**Figure 1:**
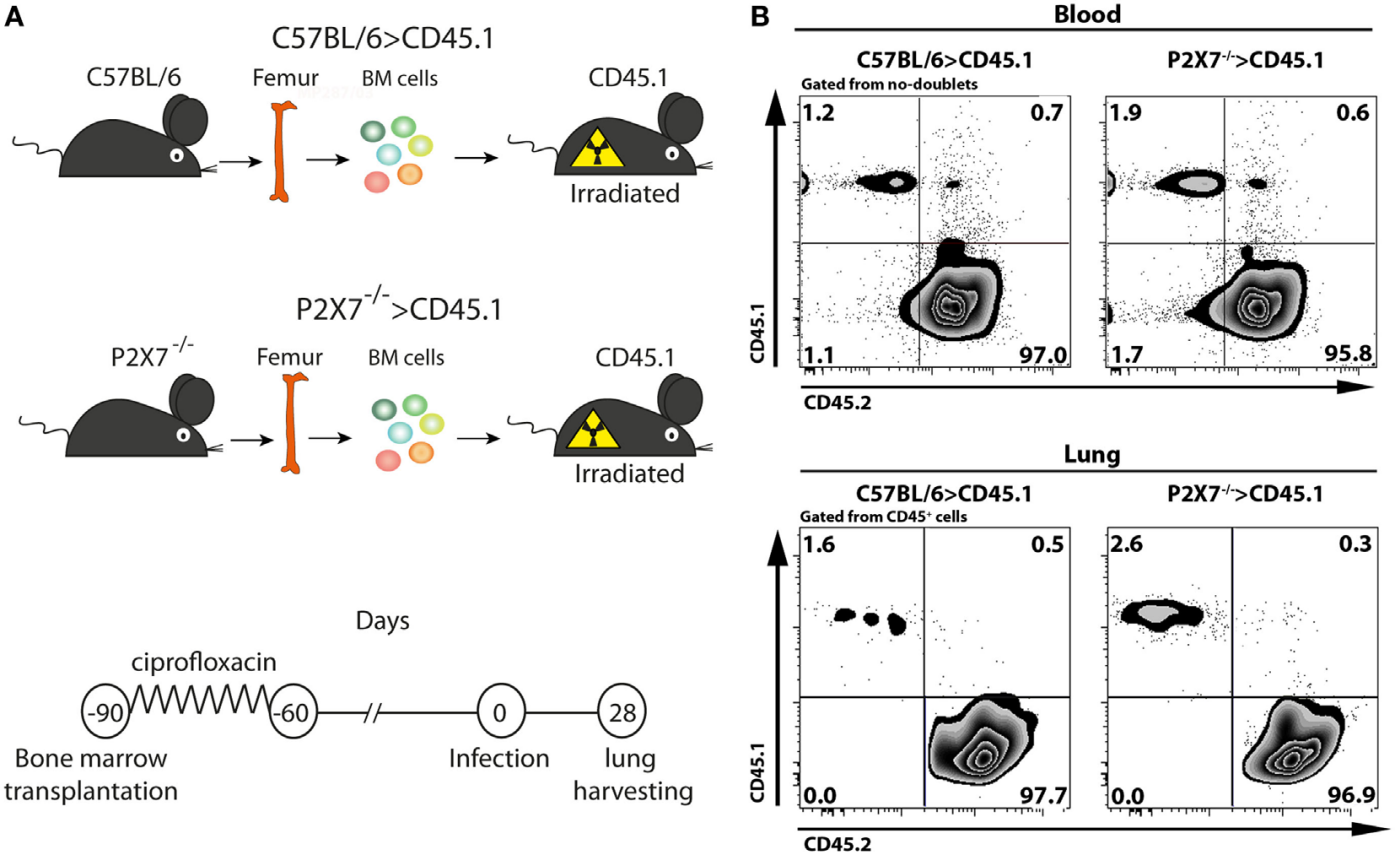
**Generation and infection of chimeric C57BL/6>CD45.1 and P2X7^−/−^>CD45.1 mice**. **(A)** Schematic illustration shows the experimental protocol. Bone marrow (BM) cells from C57BL/6 and P2X7^−/−^ mice were adoptively transferred to lethally irradiated CD45.1 mice. Ninety days later, chimeric mice were infected i.t. with approximately 100 MP287/03 bacilli. The lungs were harvested 28 days after infection. **(B)** Contour plots show CD45.1 and CD45.2 expression in blood cells and lung CD45^+^ cells from chimeric mice at 90 days post-transfer.

**Figure 2:**
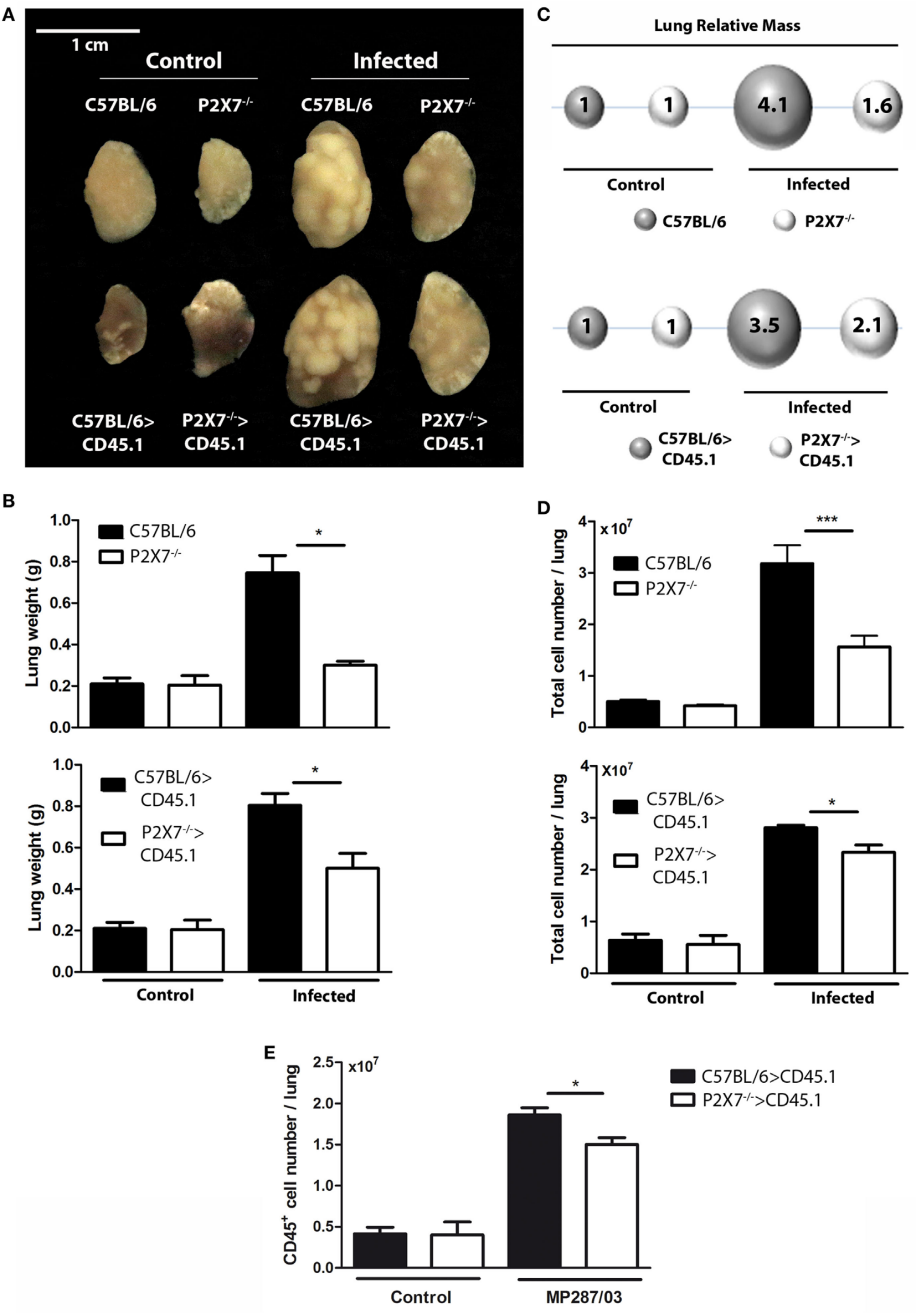
**Lung gross pathology in chimeric and non-chimeric mice on day 28 p.i., C57BL/6, P2X7^−/−^, C57BL/6>CD45.1, and P2X7^−/−^>CD45.1 mice were infected with MP287/03 bacilli**. Non-infected mice were used as controls. **(A)** Representative images of the right lungs are shown (bar scales correspond to 1 cm). **(B)** Right lung weights and **(C)** lung relative masses (circles) were evaluated. The lung relative masses were calculated by the ratios of the mean values of the lung weights in the indicated groups and the control group. **(D)** Numbers of total cells in the lungs are shown. Significant differences were observed for the indicated groups (*p < 0.05 and ***p < 0.001). The data are representative of three separate experiments with three to five mice each (means ± SEM). **(E)** Numbers of CD45^+^ cells in the lungs are shown.

To determine whether P2X7 receptor in BM-derived cells is responsible for the deleterious role of this receptor in severe TB, hematopoietic cells from C57BL/6 and P2X7^−/−^ mice were transferred into irradiated CD45.1 mice (Figure 1A). After 90 days, chimeric C57BL/6>CD45.1 and P2X7^−/−^>CD45.1 mice showed high levels of BM-derived cell reconstitution in the blood and lungs (over 95% of the CD45^+^ cells) (Figure 1B). These chimeric mice were then infected intratracheally (i.t.) with ~100 MP287/03 bacilli. We used this mycobacterial strain because it is more aggressive than Beijing 1471 strain, evidencing more clearly the effects of P2X7 receptor (13). Recapitulating the observations in infected C57BL/6 and P2X7^−/−^ mice, the lung tuberculous nodules were visually more numerous and protuberant in C57BL/6>CD45.1 mice than in P2X7^−/−^>CD45.1 mice at 28 days post-infection (p.i.) (Figure 2A). Accordingly, lung weight, lung relative mass, and cellularity were higher in infected C57BL/6 and C57BL/6>CD45.1 mice compared to P2X7^−/−^ and P2X7^−/−^>CD45.1 counterparts, respectively (Figures 2B–D). In addition, the number of CD45^+^ cells was also higher in C57BL/6>CD45.1 mice than in P2X7^−/−^>CD45.1 mice (Figure 2E).

### P2X7 Receptor in BM-Derived Cells Enhances Lung Pathology, Lung Bacterial Burden, and Bacterial Dissemination to the Liver and Spleen in Severe TB

**Figure 3:**
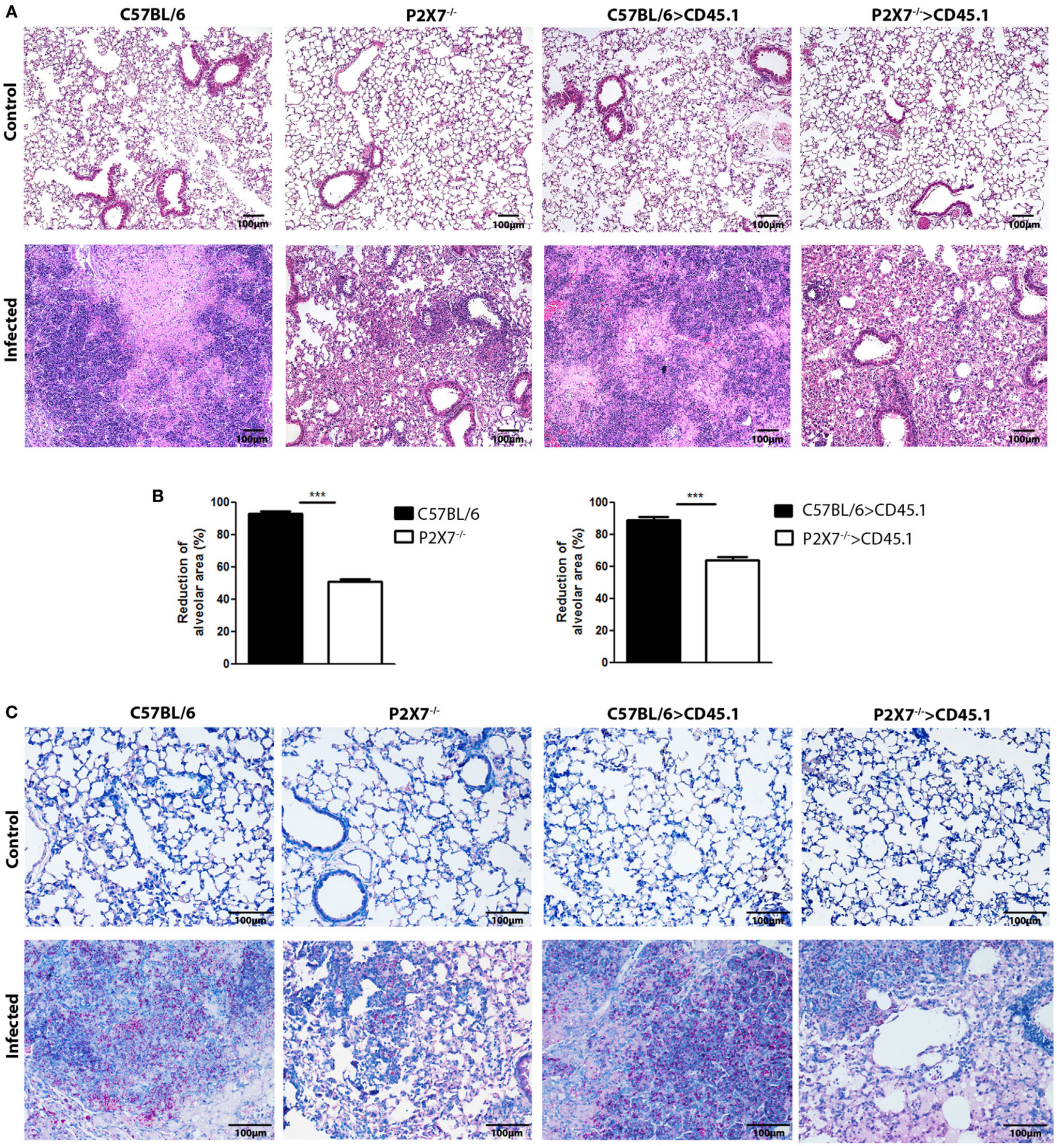
**Lung histopathology in chimeric and non-chimeric mice on day 28 p.i. C57BL/6, P2X7^−/−^, C57BL/6>CD45.1, and P2X7^−/−^>CD45.1 mice were infected with MP287/03 bacilli**. Non-infected mice were used as controls. **(A)** Images show representative lung sections stained with hematoxylin–eosin method (100 × magnification; bar scales correspond to 100 µm). **(B)** Morphometric quantification of lung sections is shown. **(C)** Images show stained with Ziehl–Neelsen method (200 × magnification; bar scales correspond to 100 µm). Significant differences were observed for the indicated groups (***p < 0.001). The data are representative of three separate experiments with three to five mice each (means ± SEM).

**Figure 4:**
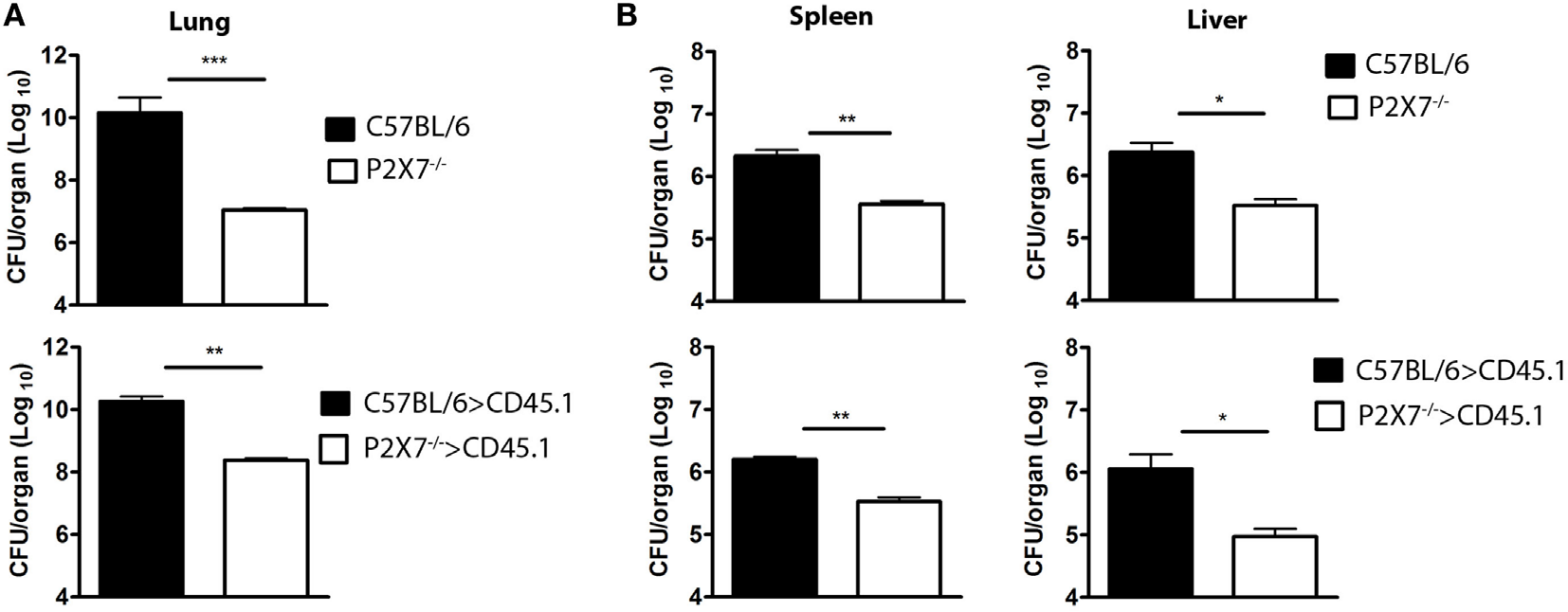
**Bacterial loads in the lungs, spleen, and liver of chimeric and non-chimeric mice on day 28 p.i. C57BL/6, P2X7^−/−^, C57BL/6>CD45.1, and P2X7^−/−^>CD45.1 mice were infected with MP287/03 bacilli**. Numbers of colony-forming units (CFUs) in the lungs **(A)**, spleen, and liver **(B)** are shown. Significant differences were observed for the indicated groups (*p < 0.05, **p < 0.01, and ***p < 0.001). The data are representative of three separate experiments with three to five mice each (means ± SEM).

Consistent with lung morphology, the histological analysis of hematoxylin–eosin (HE) stained tissue sections revealed a more severe disease in infected mice expressing the P2X7 receptor in BM-derived cells (Figure 3A). On day 28 p.i., C57BL/6 and C57BL/6>CD45.1 mice showed intense pulmonary inflammation with intra-alveolar spaces containing widespread cellular infiltrates accompanied by necrotic tissue injury. In contrast, limited cellular infiltrates and no sign of necrosis were observed in infected P2X7^−/−^ and P2X7^−/−^>CD45.1 mice. Accordingly, the areas of alveolar space were significantly lower in infected C57BL/6 and C57BL/6>CD45.1 mice compared to infected P2X7^−/−^ and P2X7^−/−^>CD45.1 mice (Figure 3B). In addition, Ziehl–Neelsen staining revealed the massive presence of acid-alcohol-resistant bacillus (BAARs) in the lungs of infected C57BL/6 and C57BL/6>CD45.1 mice, whereas less bacilli were observed in P2X7^−/−^ and P2X7^−/−^>CD45.1 counterparts, respectively (Figure 3C). Compatibly, the numbers of colony-forming units (CFUs) were higher in the lungs of infected mice expressing the P2X7 receptor in BM-derived cells (Figure 4A). Moreover, infected C57BL/6 and C57BL/6>CD45.1 mice showed more bacillus dissemination to the liver and spleen than P2X7^−/−^ and P2X7^−/−^>CD45.1 counterparts (Figure 4B). These results confirm the important role of P2X7 receptor in BM-derived cells in defining the increased resistance of P2X7^−/−^ mice to severe TB.

### P2X7 Receptor in BM-Derived Cells Leads to Enrichment of Ly6G^int^ Cells into the Lungs during Severe TB

**Figure 5:**
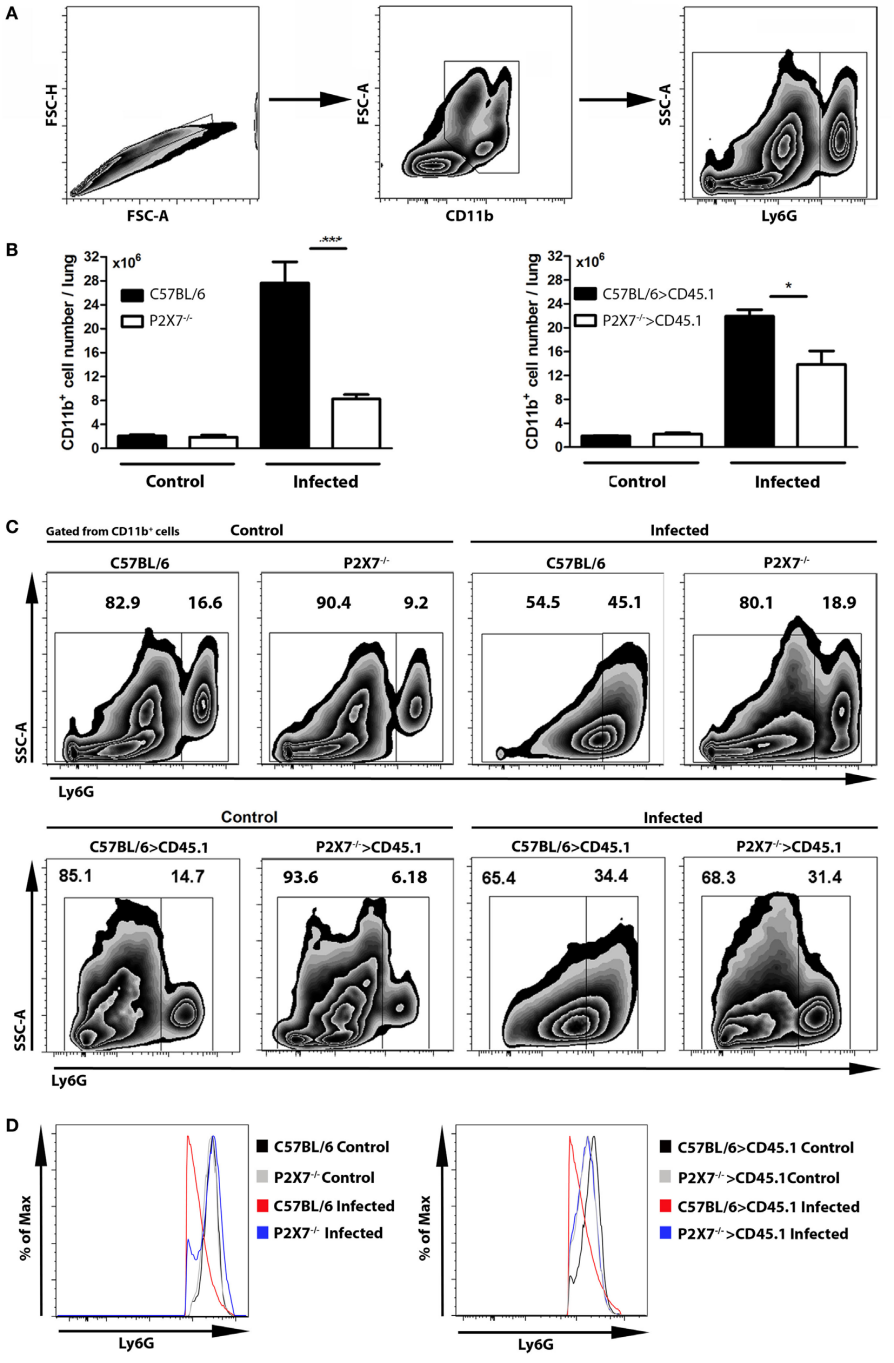

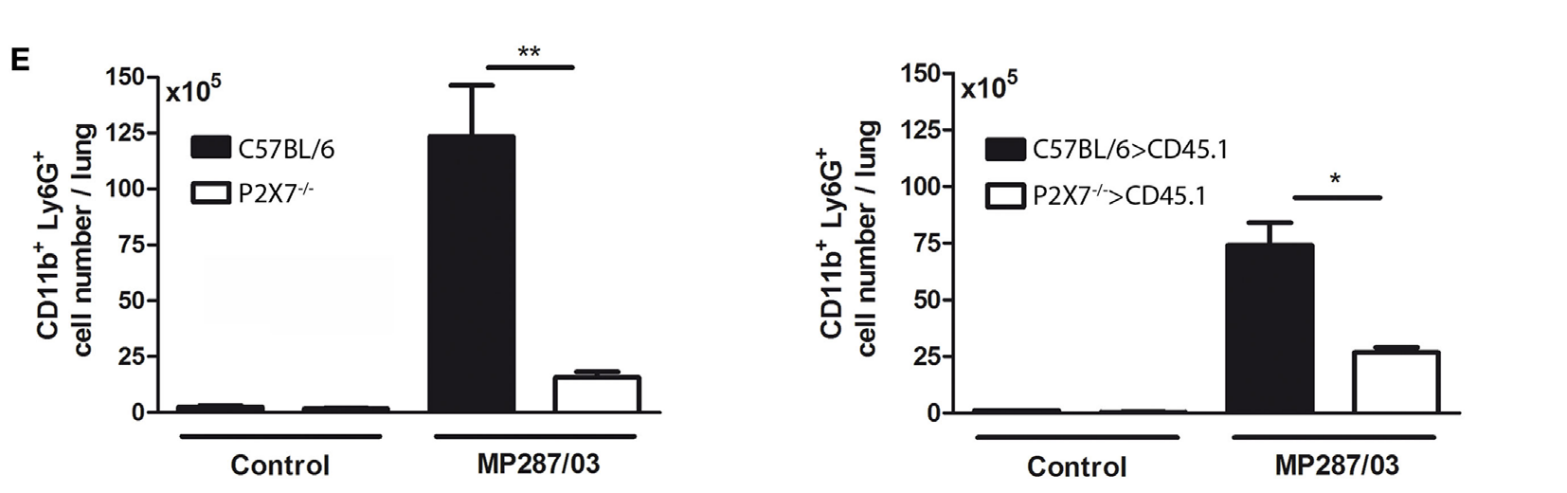
**Phenotypic profile of lung neutrophils in chimeric and non-chimeric mice on day 28 p.i. C57BL/6, P2X7^−/−^, C57BL/6>CD45.1, and P2X7^−/−^>CD45.1 mice were infected with MP287/03 bacilli**. Non-infected mice were used as controls. **(A)** Contour plots show the gate strategy used to analyze lung neutrophils. **(B)** CD11b^+^ cell numbers in the lungs are shown. **(C)** Contour plots show Ly6G expression and side scatter in lung CD11b^+^ cells. **(D)** Expression of Ly6G molecule and **(E)** numbers of Ly6G^+^ cells in the lungs are shown. The cell numbers in C57BL/6 and C57BL/6>CD45.1 mice were not calculated (ND, not done), as lung CD11b^+^ cells express intermediate levels of Ly6G. Significant differences were observed for the indicated groups (*p < 0.05 and ***p < 0.001). The data are representative of three separate experiments with three to five mice each (means ± SEM).

As a hallmark of severe TB is the presence of massive neutrophil infiltrates in the lungs (26–28), we investigated whether the absence of P2X7 receptor in BM-derived cells influences the pulmonary myeloid cell populations in MP287/03-infected chimeric mice. On day 28 p.i., C57BL/6 and C57BL/6>CD45.1 mice showed higher numbers of CD11b^+^ cells compared to P2X7^−/−^ and P2X7^−/−^>CD45.1 mice, respectively (Figures 5A,B). Furthermore, an immature-like cell population expressing intermediate levels of Ly6G predominated in infected C57BL/6 and C57BL/6>CD45.1 mice, whereas infected P2X7^−/−^ and P2X7^−/−^>CD45.1 mice presented a typical neutrophil Ly6G^high^ population (Figures 5C–E). These data indicate that P2X7 expression in BM-derived cells contributes to TB severity, which was characterized by the predominance of immature-like myeloid cells infiltrating the lungs.

## Discussion

We have previously shown the deleterious role of P2X7 receptor in severe TB caused by Beijing 1471 and MP287/03 bacilli (13). *In vitro*, these hypervirulent mycobacteria induce macrophage death by a P2X7-dependent mechanism that facilitates bacillus release. Based on these findings, we proposed that the fast intracellular multiplication of hypervirulent mycobacteria causes widespread destruction of infected macrophages. Consequently, high amounts of eATP activate the P2X7 receptor and facilitate the development of the necrotic process by cooperating with mycobacterial components exhibiting the membrane-lysing activity. This process leads to a vicious cycle that exacerbates pneumonia, lung damage, and bacillus dissemination.

*In vivo*, various cell populations can contribute to the deleterious role of P2X7 receptor in severe TB, as this receptor is expressed in many BM-derived cells (i.e., monocytes, macrophages, neutrophils, and T cells) and lung structural cells (i.e., alveolar epithelial type I cells, lung endothelial cells, and fibroblasts) (19, 22–25, 29, 30). By analyzing chimeric C57BL/6>CD45.1 and P2X7^−/−^>CD45.1 mice infected with MP287/03 bacilli, we show here that the absence of P2X7 receptor in BM-derived cells recapitulates the TB progression observed in mice lacking this receptor. According to all parameters analyzed in this study, P2X7^−/−^>CD45.1 mice developed a less severe TB compared to C57BL/6>CD45.1 mice. Infected mice lacking the P2X7 receptor in BM-derived cells showed lower lung bacterial load accompanied by attenuated pneumonia and no sign of lung necrosis. Bacterial dissemination to spleen and liver was also reduced in P2X7^−/−^>CD45.1 mice compared to C57BL/6>CD45.1 mice. Furthermore, a typical Ly6G^high^ neutrophil population infiltrated the lungs of infected P2X7^−/−^>CD45.1 mice, whereas an immature-like myeloid cell population displaying a Ly6G^int^ phenotype predominated in infected C57BL/6>CD45.1 mice.

These results are in line with our model in which P2X7 receptor of infected macrophages is decisive to aggravate the disease (11, 12). Yet, the participation of other BM-derived cell population is still an open possibility. Although neutrophils play an important role in host defense against bacterial infections, their involvement in TB is controversial (27, 28, 31). The excessive accumulation of neutrophils in the lungs is very harmful and usually associated with tissue damage during severe TB (27, 31). In addition, immature myeloid cells, mainly neutrophil precursors, are the main population infiltrating the lungs at advanced TB stages (32, 33). Myeloid cells with an immature phenotype can behave like myeloid-derived suppressor cells and make the disease worse by suppressing the immune response (32–34). This population has a CD11b^+^GR1^int^ phenotype and expresses intermediate levels of Ly6G (32). Therefore, the accumulation of Ly6G^int^ cells in the lungs of MP287/03-infected mice could be a secondary consequence of the excessive tissue damage resulting from P2X7 signaling.

Recently, it has been shown that neutrophils express the P2X7 receptor, which once activated by ATP, leads to K^+^ efflux and, consequently, to NLRP3 inflammasome activation and IL-1β secretion (35). However, the detrimental effect of P2X7 receptor during severe TB appears to be independent of NLRP3 inflammasome. The absence of NLRP3, ASC, and caspase-1 does not change TB progression in MP287/03-infected mice (data not shown). Moreover, differently from macrophages, P2X7 engagement does not induce neutrophil lysis (35). Therefore, it is unlikely that P2X7 receptor mediates lung injury by inducing neutrophil death. Alternatively, it has been shown that P2X7 activation induced by antibacterial protein LL-37 leads to suppression of spontaneous apoptosis in neutrophils (36). As neutrophil apoptosis limits the release of proinflammatory mediators and cytotoxic metabolites (37), it is possible that, in severe TB, prolongation of neutrophil life span mediated by P2X7 receptor could amplify the proinflammatory response and secondarily promote tissue injury.

In conclusion, this study helps to improve the knowledge concerning the critical role of P2X7 receptor in severe TB by demonstrating the importance of P2X7 receptor in BM-derived cells. This finding brings us a step forward in understanding the pathophysiology of aggressive forms of TB and reinforces the P2X7 receptor as a potential target for new therapeutic approaches to ameliorate the disease outcome.

## Materials and Methods

### Mice

Specific pathogen-free C57BL/6, P2X7^−/−^ (B6.129P2-P2rx7tm1Gab/J), and CD45.1 (B6.SJL-Ptprca Pepcb/BoyJ) male mice (The Jackson Laboratory, USA; generated by Pfizer Inc.) were bred at the Animal Facility of the Biomedical Science Institute, USP. Six- to eight-week-old mice were infected and maintained in microisolator cages at the Biosafety Level 3 Mice Facilities at the Faculty of Pharmaceutical Sciences, USP, under controlled temperature and humidity and were fed *ad libitum*.

### Mycobacteria

Dr. José Soares Ferreira Neto (Veterinary Medicine Institute, USP) provided the bovine *M. bovis* isolate (MP287/03—SB0295 spoligotyping). Mycobacteria were cultured in Middlebrook 7H9 medium (Difco, BD Biosciences, USA) with 0.4% sodium pyruvate (Sigma-Aldrich, USA), 0.05% Tween 80 (Sigma-Aldrich), and 10% ADC (albumin–dextrose–catalase; Difco). Frozen aliquots of 10^8^ bacilli/ml, at –80°C, were thawed and cultured in complete medium for 7 days at 37°C. The bacilli were sonicated for 1 min, homogenized and maintained for 10 min at rest to prevent bacterial clumps, which were monitored by microscopic examination. The bacterial concentrations were determined by spectrophotometry at 600 nm.

### CFU Counting

The mycobacterial burden was quantified by sequential dilutions and the culture of tissue homogenates (lung, spleen, and liver) in Middlebrook 7H10 medium (Difco) with 0.4% sodium pyruvate and 10% OADC (oleic acid–albumin–dextrose–catalase; Difco). Three weeks after incubation at 37°C, the CFU numbers were determined.

### Mouse Infection

After anesthetizing mice with xylazine (Vetbrands, Brazil; 15 mg/kg) and ketamine (Vetbrands, 100 mg/kg), a volume of 60 µl of the mycobacterial suspension (~100 bacilli) was introduced in the trachea through a short midline incision, which was then sutured with sterile silk (38).

### Lethal Irradiation and BM Reconstitution

Bone marrow cells were harvest from femur of C57BL/6 or P2X7^−/−^ mice by flushing with PBS. A single-cell preparation was obtained by carefully cycling through a 26-gauge needle. Recipient CD45.1 mice were irradiated with a dose of 12 Gy from a ^137^Cs source. After irradiation, 2 × 10^7^ BM cells from C57BL/6 and P2X7R^−/−^ mice in a volume of 200 µl PBS were transferred i.v. under anesthesia. The chimeric mice were housed for at least 12 weeks before infection and were fed with water containing antibiotic (0.1 mg/ml of ciprofloxacin) in the first 4 weeks after BM transplantation.

### Macroscopic and Microscopic Analysis of the Lungs

Lung relative mass was calculated (infected mouse lung weight/control mouse lung weight). The superior lobes of the right lungs were fixed with 10% buffered formalin, photographed, and embedded in paraffin. Serial 4–5 µm sections were stained with HE dye to analyze the tissue alterations and by the Ziehl–Neelsen method to detected BAARs. The samples were examined with a Leica microscope (Germany), and images were captured with a Coolpix P995 Nikon camera (Japan).

### Morphometric Analysis of Lung Tissue

The reduction in the percentages of pulmonary intralveolar space was determined as described elsewhere (13, 39). Eight random images of each lung HE-stained section (100× magnification) were analyzed using the ImageJ software (National Institutes of Health, USA).

### Cell Phenotypic Analysis of Lung Infiltrates

The left lungs were dissected and digested with Collagenase type 4 (Sigma-Aldrich; 0.5 mg/ml) at 37°C for 40 min. A syringe plunger (BD Bioscience) was used to disperse the cells. Cell suspensions were then filtered with a cell strainer (Corning Inc., USA) and incubated with ACK lysing Buffer (Thermo Fisher Scientific) at room temperature for 1 min to deplete erythrocytes. Cells (1 × 10^6^) were stained using appropriate combinations of FITC-, PercP-, Pecy7-, and APC-labeled monoclonal antibodies to CD11b (M1/70), CD45.1 (A20), Ly6G (1A8) (BD Pharmingen, USA), and CD45.2 (104) (eBioscience). Cells were fixed with 2% paraformaldehyde and analyzed by flow cytometry (FACSCanto, BD Biosciences) using the FlowJo software.

### Statistical Analysis

Data were statistically analyzed by Mann–Whitney test with the GraphPad Prism 5 software (GraphPad, USA) and were considered significantly different when *p* < 0.05 (5%).

## Ethics Statement

All procedures were in accordance with the national regulations of the National Board of Health and Brazilian College of Animal Experimentation (COBEA, Brazil), with respect to their ethical guidelines for mouse experimentation and welfare. The protocol was approved by the Animal Care Committee of the Biomedical Science Institute, University of São Paulo, with permit number 153/11.

## Author Contributions

CCB, EA, and MD-L designed and conceived the experiments, analyzed the data, and wrote the manuscript. CCB, EA, AC, ES, and RN performed the experiment. MH, JA, and EL contributed with reagents, materials, and analysis tools.

## Conflict of Interest Statement

The authors declare that the research was conducted in the absence of any commercial or financial relationships that could be construed as a potential conflict of interest.
